# Supplementary material for: Genetic variation of the mitochondrial DNA control region across plains bison herds in USA and Canada
Source: PLoS One. 2022 Mar 10;17(3):e0264823. doi: 10.1371/journal.pone.0264823 (PMC8912233; doi:10.1371/journal.pone.0264823)
Supplement: S1 Table — Polymorphisms were determined by alignment with the reference mitochondrial genome GU946990. (DOCX) [file pone.0264823.s001.docx]

| **DOI & PCA mtDNA haplotype** | **Single nucleotide polymorphisms (SNPs)** |
| --- | --- |
| **Hap 1/0** | no SNPs |
| **Hap 1/1** | 351delG |
| **Hap 1/2** | 15952delG, 351delG |
| **Hap 1/3** | 15952delG, 221.1C, 351delG |
| **Hap 1/4** | 15952.1G, 15955delA |
| **Hap 1/5** | 16248delG, 351delG |
| **Hap 1/6** | 16235.1A |
| **Hap 1/7** | 221.1C |
| **Hap 2/1** | 15952delG, 16248G→A, 351delG |
| **Hap 2/2** | 16248G→A, 351delG |
| **Hap 3** | 15895C→T, 15952delG, 15955delA, 16122C→T, 16189T→C, 16283A→G, 166A→G, 351delG |
| **Hap 4/1** | 15895C→T, 15952delG, 15955delA, 16040C→T, 16050C→T, 16122C→T, 16131T→C, 16283A→G |
| **Hap 4/2** | 15895C→T, 15952delG, 15955delA, 16040C→T, 16050C→T, 16122C→T, 16131T→C, 16283A→G, 221.1C |
| **Hap 5/1** | 15895C→T, 15952delG, 15955delA, 16122C→T, 16283A→G, 166A→G, 221.1C, 351delG |
| **Hap 5/2** | 15895C→T, 15952delG, 15955delA, 16122C→T, 16283A→G, 166A→G, 351delG |
| **Hap 6** | 15895C→T, 15952delG, 15955delA, 16122C→T, 8G→A, 201delA, 351delG |
| **Hap 7** | 15895C→T, 15952delG, 15957A→G, 15965C→T, 16042T→C, 16122C→T, 16283A→G, 351delG |
| **Hap 8** | 16041C→T, 221.1C |
| **Hap 9** | 16283A→G, 351delG |
| **Hap 10/1** | 16122C→T, 351delG |
| **Hap 10/2** | 15952delG, 15955delA, 16122C→T |
| **Hap 11/1** | 15895C→T, 15952delG, 15955delA, 16122C→T, 16279C→T, 351delG |
| **Hap 11/2** | 15895C→T, 15932.1A, 15952delG, 15955delA, 16122C→T, 16279C→T, 351delG |
| **Total # of haplotypes** | 23 |
