# Supplementary material for: Genetic variation of the mitochondrial DNA control region across plains bison herds in USA and Canada
Source: PLoS One. 2022 Mar 10;17(3):e0264823. doi: 10.1371/journal.pone.0264823 (PMC8912233; doi:10.1371/journal.pone.0264823)
Supplement: S2 Table — (DOCX) [file pone.0264823.s002.docx]

| **DOI & PCA mtDNA haplotype** | **BADL** | **BOOK** | **ELK** | **FTN** | **GRASS** | **NBR** | **NER** | **NSM** | **RMA** | | **TAPR** | **WICA** | **WM** | **WRST** | **THRO** |
| --- | --- | --- | --- | --- | --- | --- | --- | --- | --- | --- | --- | --- | --- | --- | --- |
| **Hap 1/0** | 15 | 2 |  | 10 |  |  | 14 |  | |  | 2 | 3 | 3 |  | 13 |
| **Hap 1/1** |  | 4 | 12 | 2 | 2 | 7 |  | 12 | | 5 | 11 | 9 | 4 | 7 |  |
| **Hap 1/2** |  | 1 |  |  |  |  |  |  | |  |  |  | 3 |  |  |
| **Hap 1/3** |  |  |  |  |  |  |  |  | |  | 1 |  |  |  |  |
| **Hap 1/4** |  |  |  |  |  |  |  |  | |  |  |  |  |  | 1 |
| **Hap 1/5** |  |  |  |  |  |  |  |  | |  | 1 |  |  |  |  |
| **Hap 1/6** |  |  |  |  |  |  | 1 |  | |  |  |  |  |  |  |
| **Hap 1/7** |  | 1 |  |  |  |  |  |  | |  |  |  | 1 |  | 1 |
| **Hap 2/1** |  | 2 |  |  |  |  |  |  | |  |  |  |  |  |  |
| **Hap 2/2** |  | 1 |  |  |  |  |  |  | |  |  |  |  |  |  |
| **Hap 3** |  | 1 |  |  |  |  |  |  | |  |  |  |  |  |  |
| **Hap 4/1** |  | 2 |  |  |  |  |  |  | |  |  |  |  |  |  |
| **Hap 4/2** |  | 1 |  |  |  |  |  |  | |  |  |  |  |  |  |
| **Hap 5/1** |  |  | 1 |  | 7 |  |  | 2 | |  |  |  |  | 5 |  |
| **Hap 5/2** |  |  | 2 |  | 3 | 5 |  | 1 | | 4 |  |  | 2 |  |  |
| **Hap 6** |  |  |  | 2 |  | 2 |  |  | | 3 |  |  |  | 3 |  |
| **Hap 7** |  |  |  | 1 |  | 1 |  |  | | 2 |  |  |  |  |  |
| **Hap 8** |  |  |  |  | 3 |  |  |  | |  |  |  |  |  |  |
| **Hap 9** |  |  |  |  |  |  |  |  | | 1 |  |  |  |  |  |
| **Hap 10/1** |  |  |  |  |  |  |  |  | |  |  | 1 |  |  |  |
| **Hap 10/2** |  |  |  |  |  |  |  |  | |  |  | 1 |  |  |  |
| **Hap 11/1** |  |  |  |  |  |  |  |  | |  |  |  | 1 |  |  |
| **Hap 11/2** |  |  |  |  |  |  |  |  | |  |  |  | 1 |  |  |
| **Total # of animals** | 15 | 15 | 15 | 15 | 15 | 15 | 15 | 15 | | 15 | 15 | 14 | 15 | 15 | 15 |

BADL, Badlands National Park; BOOK, Book Cliffs; ELK, Elk Island National Park; FTN, Fort Niobrara National Wildlife Refuge; GRASS, Grasslands National Park; NBR, National Bison Range; NER, National Elk Refuge; NSM, Neal Smith National Wildlife Refuge; RMA, Rocky Mountain Arsenal National Wildlife Refuge; TAPR, Tallgrass Prairie National Preserve; WICA, Wind Cave National Park; WM, Wichita Mountains Wildlife Refuge; WRST, Wrangell St. Elias National Park and Preserve; THRO, Theodore Roosevelt National Park.
