# Supplementary material for: Genetic variation of the mitochondrial DNA control region across plains bison herds in USA and Canada
Source: PLoS One. 2022 Mar 10;17(3):e0264823. doi: 10.1371/journal.pone.0264823 (PMC8912233; doi:10.1371/journal.pone.0264823)
Supplement: S3 Table — (DOCX) [file pone.0264823.s003.docx]

| **Source of Variation** | **Percent of Variation** | **p-value** |
| --- | --- | --- |
| Among Populations | 21.41 | <0.0001 |
| Within Populations | 78.59 | <0.0001 |
