# Supplementary material for: Genetic variation of the mitochondrial DNA control region across plains bison herds in USA and Canada
Source: PLoS One. 2022 Mar 10;17(3):e0264823. doi: 10.1371/journal.pone.0264823 (PMC8912233; doi:10.1371/journal.pone.0264823)
Supplement: S4 Table — (DOCX) [file pone.0264823.s004.docx]

| **Haplotypes in Douglas et al., 2011 [12]** | **Sample** | **SNPs in the control region vs. GU946990 (bHap10)** | **DOI & PCA haplotypes without indels** | **DOI & PCA sub-haplotypes with indels** | **Herd in Douglas et al., 2011 [12]** | **Other Douglas et al., 2011 [12] herds having the same haplotype** |
| --- | --- | --- | --- | --- | --- | --- |
| **bHap2** | GU947001 | 351delG | Hap 1 | Hap 1/1 | National Bison Range, Montana | Private herd in Montana |
| **bHap3** | GU946992 | 15895C→T, 15952delG, 15955delA, 16122C→T, 16189T→C, 16283A→G, 166A→G, 351delG | Hap 3 | Hap 3 | Private herd in Montana |  |
| **bHap4** | GU946980 | 15895C→T, 15952delG, 15955delA, 16122C→T, 16283A→G, 166A→G, 351delG | Hap 5 | Hap 5/2 | Private herd in Montana |  |
| **bHap5** | GU946982 | 15895C→T, 15952delG, 15955delA, 16122C→T, 16283A→G, 166A→G, 351delG | Hap 5 | Hap 5/2 | Private herd in Montana |  |
| **bHap6** | GU946985 | 15895C→T, 15952delG, 15955delA, 16122C→T, 16283A→G**, 16290C→T,** 166A→G, 221.C, 351delG | not found | not found | Private herd in Montana |  |
| **bHap7** | GU946987 | 15895C→T, 15952delG, 15955delA, **16048G→A, 16105C→T, 16110G→A,** 16122C→T, 16283A→G, 166A→G, **202C→T**, 351delG | not found | not found | Private herd in Montana |  |
| **bHap8** | GU946988 | none | Hap 1 | Hap 1/0 | Private herd in Montana |  |
| **bHap9** | GU946997 | 15895C→T, 15952delG, 15955delA, 16122C→T, 16283A→G, 166A→G, 351delG | Hap 5 | Hap 5/2 | Private herd in Montana |  |
| **bHap10** | GU946990 | none | Hap 1 | Hap 1/0 | Private herd in Montana | Fort Niobrara NWR |
| **bHap11** | GU946994 | none | Hap 1 | Hap 1/0 | Private herd in Montana |  |
| **bHap12** | GU946998 | 15895C→T, 15952delG, 15955delA, 16122C→T, 16189T→C, 16283A→G, 166A→G | Hap 3 | Hap 3 w/ 351delG | Private herd in Montana |  |
| **bHap13** | GU947002 | 15895C→T, 15952delG, 15957A→G, 15965C→T, 16042T→C, 16122C→T, 16283A→G, 351delG | Hap 7 | Hap 7 | Texas State Bison herd | Private herd in Montana |
| **wHap14** | GU947006 | 15895C→T, 15952delG, 15955delA, 16050C→T, 16122C→T, 16131T→C, **16240C→T**, 351delG | not found | not found | Elk Island NP (*B. bison athabascae*) |  |
| **wHap15** | GU947005 | 15895C→T, 15952delG, 15955delA, 15965C→T, 16122C→T, **16148T→C**, 351delG | not found | not found | Elk Island NP (*B. bison athabascae*) |  |
| **bHap16** | GU947003 | 15895C→T, 15952delG, 15957A→G, 15965C→T, 16042T→C, 16122C→T, 16283A→G, 351delG | Hap 7 | Hap 7 | Texas State Bison herd |  |
| **bHap17** | GU947004 | 221.C, 351delG | Hap 1 | Hap 1/1 + Hap 1/7 | Yellowstone NP |  |

Red SNPs are not found in the DOI & PCA herds in this study.
